# A novel 4-aminoquinazoline derivative, DHW-208, suppresses the growth of human breast cancer cells by targeting the PI3K/AKT/mTOR pathway

**DOI:** 10.1038/s41419-020-2690-y

**Published:** 2020-06-30

**Authors:** Shu Wang, Yingshi Zhang, Tianshu Ren, Qiong Wu, Hongyuan Lu, Xiaochun Qin, Yuyan Liu, Huaiwei Ding, Qingchun Zhao

**Affiliations:** 1 Department of Life Science and Biochemistry, Shenyang Pharmaceutical University, 110016 Shenyang, China; 2 Department of Pharmacy, General Hospital of Northern Theater Command, 110840 Shenyang, China; 3 Key Laboratory of Structure-Based Drug Design and Discovery of Ministry of Education, Shenyang Pharmaceutical University, 110016 Shenyang, China

**Keywords:** Breast cancer, Breast cancer, Targeted therapies, Targeted therapies, Apoptosis

## Abstract

Breast cancer is one of the most frequent cancers among women worldwide. However, there is still no effective therapeutic strategy for advanced breast cancer that has metastasized. Aberrant activation of the PI3K/AKT/mTOR pathway is an essential step for the growth of human breast cancers. In our previous study, we designed and synthesized DHW-208 (2,4-difluoro-N-(5-(4-((1-(2-hydroxyethyl)-1H-pyrazol-4-yl)amino)quinazolin-6-yl)-2-methoxypyridin-3-yl)benzenesulfonamide) as a novel pan-PI3K inhibitor. This study aimed to assess the therapeutic efficacy of DHW-208 in breast cancer and investigate its underlying mechanism. We found that DHW-208 inhibited the growth, proliferation, migration, and invasion of breast cancer cells. Moreover, DHW-208 induced breast cancer cell apoptosis via the mitochondrial pathway and induced G0/G1 cell-cycle arrest. In vitro results show that DHW-208 is a dual inhibitor of PI3K and mTOR, and suppress the growth of human breast cancer cells by targeting the PI3K/AKT/mTOR pathway. Consistent with the in vitro results, in vivo studies demonstrated that DHW-208 elicits an antitumor effect by inhibiting the PI3K/AKT/mTOR-signaling pathway with a high degree of safety in breast cancer. Above all, we report for the first time that DHW-208 suppressed the growth of human breast cancer cells by inhibiting the PI3K/AKT/mTOR-signaling pathway both in vivo and in vitro. Our study may provide evidence for the use of DHW-208 as an effective, novel therapeutic candidate for the treatment of human breast cancers in clinical trials.

## Introduction

Improvements in the detection and treatment of breast cancer have led to better prognosis and survival, with a 5-year survival rate of nearly 90%^1,2^. However, breast cancer is still one of the most frequent malignant diseases in women worldwide and the second leading cause of mortality in females^1,3,4^. Moreover, there is still no effective treatment strategy for advanced breast cancer that has metastasized^3,5^. To date, many therapeutic targets have been verified for treating breast cancers, including CDK4/6 inhibitors, HDAC inhibitor, Estrogen pathway antagonists, VEGF inhibitors, PI3K inhibitors, mTOR inhibitors, etc.^6–8^. Among these, the phosphate idylinositol 3-kinase (PI3K)/AKT/mammalian target of rapamycin (mTOR) pathway was found to play a central role in the cell physiology of breast cancer^9–11^. Mutations in the PI3K/AKT/mTOR pathway are frequently detected in breast cancer. Approximately 60% of breast cancer tumors have genetic alterations that activate the PI3K/AKT/mTOR pathway. Because of the important role that the PI3K/AKT/mTOR pathway plays in tumors, many inhibitors that target this pathway have been developed^12–14^. The first PI3K inhibitor for breast tumors, Alpelisib tablets, was approved by the FDA in May 2019 for the treatment of advanced or metastatic breast cancer^15^. Despite advances in the development of drugs targeting the PI3K/AKT/mTOR pathway, much safer and more effective targeted drugs are still needed in the clinic.

The PI3K/AKT/mTOR pathway controls numerous cellular functions such as growth, proliferation, survival, motility, and metabolism^16–19^. Activated by upstream signaling molecules, phosphatidylinositol 4,5-diphosphate (PIP2) in the plasma membrane is converted by the catalytic subunit of P13K to phosphatidylinositol 3,4,5-triphosphate (PIP3). PIP3 brings phosphoinositide-dependent protein kinase 1 (PDK1) to the cell membrane where the latter phosphorylates Thr308 in the catalytic domain of AKT. Subsequently, Ser473, in the regulatory domain of AKT is phosphorylated by mammalian rapamycin complex 2 (mTORC2), and AKT kinase activity becomes fully activated^13,16,20^. Activated mTORC1 can also activate ribosomal protein S6 kinase (S6K) and eukaryotic translation initiation factor 4E-binding protein (4EBP), promoting protein synthesis and cell proliferation^21–23^. The roles AKT played in the cell are numerous and various, but all result in anti-apoptosis, or pro-cell proliferation effects^24^. The physiological functions of AKT included involvement in metabolism, protein synthesis, apoptotic pathways, cell cycle, and transcription factor regulation^25–27^. These processes are identified as key factors in establishing and maintaining oncogenic phenotypes^28,29^.

**Fig. 1:**
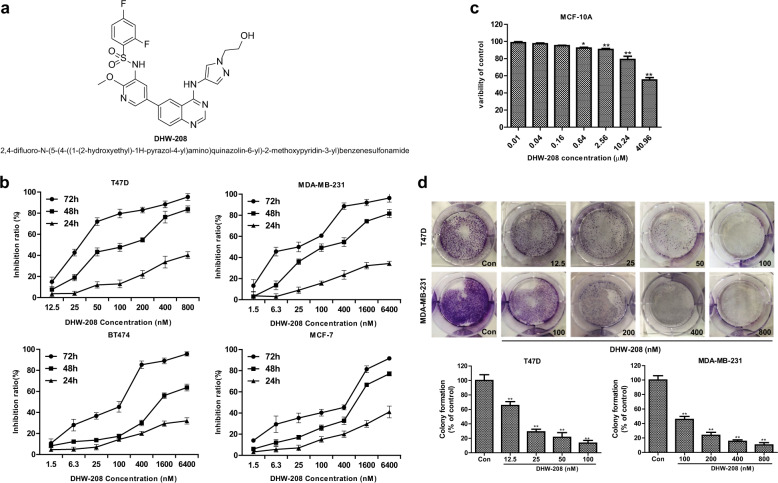
Effects of DHW-208 on T47D and MDA-MB-231 cell viability and proliferation. **a** Chemical structure of DHW-208. **b** SRB assay for cell viability of T47D, MDA-MB-231, BT474, and MCF-7 cells treated with different concentrations of DHW-208 for 24, 48, and 72 h. **c** SRB assay to determine cytotoxicity toward normal breast cell MCF-10A at 72 h. **d** Growth inhibition effects of DHW-208 on T47D and MDA-MB-231 cells were measured by colony formation assay. Bar graphs showed the quantitative results of the colony formation assay (down). Each value is the mean (±SD) from triplicate samples. *p < 0.05, **p < 0.01 vs. control.

Recently, a number of 4-amino-quinazoline derivatives have been developed as selective inhibitors of tyrosine kinase, such as Gefitinib, Erlotinib, and Lapatinib. Moreover, some 4-aminoquinazoline derivatives have ever been reported as PI3K inhibitors^30^. Previously, we had designed and synthesized a series of 4-aminoquinazoline derivatives targeting the PI3K/AKT/mTOR-signaling pathway^31^. Among them, 2,4-difluoro-N-(5-(4-((1-(2-hydroxyethyl)-1H-pyrazol-4-yl)amino)quinazolin-6-yl)-2-methoxypyridin-3-yl)benzenesulfonamide (DHW-208) (Fig. 1a) showed optimal anti-breast cancer activity and significant inhibitory activity against four main subunits of PI3K (p110α/p85α, p110β/p85α, p120γ, p110δ/p85α). In the present study, we determined the effects of DHW-208 on the growth, proliferation, migration, and invasion of breast cancer cells in vitro and those related molecular mechanism. We then studied the effect of DHW-208 on tumor growth in nude mice xenografted with human breast cancer cells.

## Materials and methods

### Reagents and antibodies

DHW-208 was synthesized by Pharmaceutical chemistry laboratory, Shenyang Pharmaceutical University, Shenyang, China. BEZ235 was obtained from MedChem Express (NJ, USA). DMEM was purchased from Hyclone (Logan, UT, USA). Antibodies for phospho-AKT (Ser473) (#9271), phospho-AKT (Thr308) (#9275), AKT (#4691), phospho-mTOR (#2971), mTOR (#2972), phospho-p70S6 kinase (#9205), p70S6 kinase (#9202), phospho-4EBP1 (#2855), 4EBP1 (#9644), Caspase-3 (#9662), Cleaved caspase-3 (#9661), Caspase-9 (#9508), Cleaved caspase-9 (#7237), PARP (#9532), Bad (#9292), Bax (#5023), bcl-2 (#4223), p21 (#2947), PCNA (#2586), Rb (#3909), p-Rb(#3908), Cyclin D1 (#2922), and β-actin (#3700) antibodies were purchased from Cell Signaling Technology (Danvers, MA, USA). The secondary antibodies were obtained from ZSGB- BIO (Beijing, China).

### Cell lines

Human breast cancer cells T47D, MDA-MB-231, BT474, MCF-7, and breast cells MCF-10A were purchased from American Type Culture Collection (ATCC, Manassas, VA, USA). All cells were cultured in DMEM supplemented with 10% fetal bovine serum (FBS) and incubated in an environment at 37 °C containing 5% CO_2_.

### Western blot analysis

RIPA buffers, including protease inhibitors, homogenized cells, and tumor tissues. Protein concentrations were determined by BCA protein detection kit. The proteins were separated by SDS–PAGE and transferred to PVDF membrane by electrophoresis. Membranes were immunoblotted using specific primary antibodies and then incubated the membrane with HRP-conjugated secondary antibody. The immune response bands were observed with the ECL assay kit. Blots were imaged by Image Quant LAS 4000 (GE Healthcare Life Sciences, Piscataway, NJ, USA).

### Immunofluorescence microscopy

Cells were inoculated into the six-well plate in each well and subjected to 24 h incubation. After being treated with DHW-208 for 24 h, the samples were fixed with 4% formaldehyde and permeabilized with 0.1% (v/v) Triton X-100. Then the samples were blocked with 5% bovine serum albumin in PBS for 30 min and incubated with p-AKT (Ser473) overnight at 4 °C. Washing with PBS twice, the samples were incubated with FITC-conjugated secondary antibody for 1 h at room temperature in the dark. DAPI staining was performed and immunofluorescence was observed by a fluorescence microscope (Olympus, Japan).

### Antiproliferative activity

Cell viability was assessed by sulforhodamine B (SRB) staining. Cells were incubated with DHW-208 with various concentrations in 96-well plates and incubated at 37 °C with 5% CO_2_ for 24, 48, and 72 h. Afterwards, the samples were fixed with 10% trichloroacetic acid solution. The optical density values were determined at 540 nm by a microplate reader (Elx 800 Bio-Tek, USA).

### Colony formation

Cells were inoculated on six-well plates, cultured overnight, and treated with DMSO or DHW-208 at different concentrations for 72 h. Then washed with PBS and cultured in full growth medium for another 10 days. The fresh medium was replaced every 3 days. After fixed with 100% methanol, the cells were stained with 0.1% crystal violet.

### Wound healing scratch assay

Sterile microtubules were used to scratch the tip of fused cells. Then cells were washed with PBS twice. The migration distance was photographed under a microscope (Olympus, Japan). Image J software was used to determine the wound area at 0 and 48 h, respectively.

### Migration and invasion assay

Cells invasion assay was measured with 24-well transwell plate (Corning Life Sciences, MA, USA). Cells in serum-free medium were seeded onto the upper chamber uncoated or coated with Matrigel (Becton Dickinson, CA, USA). The lower chamber was filled the complete medium containing 10% FBS. After 48 h, the remaining cells on the upper side of the membrane were wiped with cotton swabs. The bottom side were fixed with 4% paraformaldehyde. The cells were stained with 0.1% crystal violet and counted under a microscope (Olympus, Japan).

### Transmission electron microscopy

Cells were collected and fixed with 3% glutaraldehyde. Then the samples were postfixed with 1% OsO_4_, then dehydrated in ascending series of ethanol, embedded, and sectioned. Stained with uranyl acetate and lead citrate, the samples were observed under an H-7650 transmission electron microscope (Hitachi, Japan).

### Cell morphology analysis

After incubating with DHW-208 for 48 h, cells were stained with Hoechst 33342. After washed with PBS twice, the samples were photographed under a fluorescence microscope (Olympus, Japan).

### Annexin FITC/PI assay

After treated with DHW-208 for indicated times, cells were fixed with 70% ethanol overnight, and stained with Annexin-V FITC/PI for 30 min in the dark at room temperature before tested by fluorescence-activated cell sorting (FACS) (Becton-Dickinson, NJ, USA).

### Mitochondrial membrane potential assay

After treated with DHW-208 for indicated times, cells plated in phenol red-free growth medium were treated with 1 mg/mL JC-1 dye for 30 min at 37 °C and analyzed by FACS.

### Cell cycle assay

After treated with DHW-208 for indicated times. The samples were permeabilized and stained with rat anti-BrdU antibody and 10 μg/mL 7-amino-actinomycin D before being subjected to FACS.

### Antitumor effects in vivo

All animal studies were obtained from Beijing Vital River Laboratory Animal Technology in accordance with the guidelines of the Animal Experimental Ethics Committee of Shenyang Pharmaceutical University and complied with the internationally recognized Animal Research: Reporting of In vivo Experiments guideline. T47D cells (1 × 10^7^ cells/nude mice) suspended with serum-free media were subcutaneously injected into the right flank of female BALB/c nude mice (4–6 weeks). The tumor diameters and body weight were measured every 4 days. The tumor volumes were calculated by the formula *V* = length × width 2/2. When the tumor volumes reached to about 100 mm^3^, the mice were randomized into five groups (*n* = 6) that administered with 0.2 mL vehicle (DMSO:PEG300:saline = 1:4:5), BEZ235 (20 mg/kg), and DHW-208 (10, 20, and 40 mg/kg) by oral gavage daily for 20 days. At the end of the experiment, all tumors and organs were removed and measured.

### Hematoxylin and eosin (H&E) staining

After the nude mice were sacrificed, an autopsy was performed. The samples were fixed in 10% neutral buffered formalin. Embedded in paraffin, the tumor samples were cut into 5 μm thickness and stained with H&E. Finally, the tumor tissues were observed under a microscope (Olympus, Japan).

### Statistical analysis

GraphPad Prism 5 (GraphPad Software, CA, USA) was used for statistical analysis. One- way analysis of variance (ANOVA) was used to analyse the significance between the groups. All data are expressed as means values ± SD. *P*‐value < 0.05 was considered statistically significant.

## Results

### DHW-208 inhibits the growth, migration, and invasion of human breast cancer cells

**Fig. 2:**
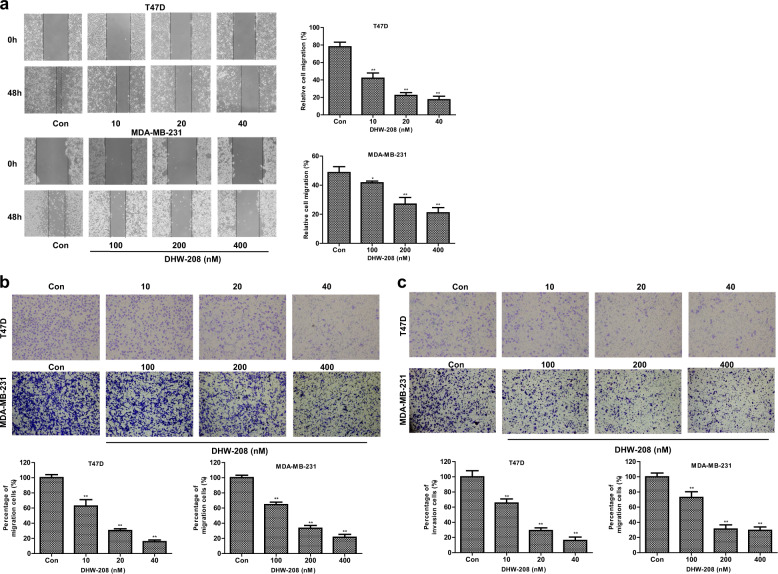
Effects of DHW-208 on T47D and MDA-MB-231 cells migration and invasion. **a** The effect of DHW-208 on cell migration of T47D and MDA-MB-231 cells was measured by wound healing assay (×100 magnification). The migration rates of T47D and MDA-MB-231 cells were calculated by the formula shown right. **b** Transwell assay was performed to assess the migration of T47D and MDA-MB-231 cells (×200 magnification). Bar graphs showed the quantitative results of the migration (down). **c** Transwell assay was performed to assess the invasion of T47D and MDA-MB-231 cells (×200 magnification). Bar graphs showed the quantitative results of the invasion (down). Each value is the mean (±SD) from triplicate samples. *p < 0.05, **p < 0.01 vs. control.

PI3K/AKT/mTOR-signaling pathway has diverse functions, including the regulation of cellular survival, proliferation, cell cycle, migration, and apoptosis. Previous studies have shown that the growth of breast tumor cells was closely related to the activation of PI3K/AKT/mTOR pathway^13^. The SRB assay showed that DHW-208 exhibits antiproliferative activity against breast cancer cells. As shown in Fig. 1b, the proliferation of breast cancer cells T47D, MDA-MB-231, BT474, and MCF-7 was inhibited by DHW-208 in a time-dependent and concentration-dependent manner. For subsequent experiments, a concentration of ~2× the IC_50_ value (40 nM for T47D and 400 nM for MDA-MB-231 cells) was used, which had similar efficiency and a stable inhibition rate in the growth-inhibition curve. The cytotoxicity of DHW-208 to normal breast cells MCF-10A was much lower than that to tumor cells, indicating the hypotoxicity of DHW-208 to normal breast cells (Fig. 2c). The long-term efficacy of DHW-208 toward both T47D and MDA-MB-231 cells was assessed by a colony formation assay, and the results showed that the DHW-208-treated T47D and MDA-MB-231 cells lost the capacity to proliferate in a concentration-dependent manner (Fig. 2d). The results of the colony formation assay were consistent with the growth-inhibitory activity noted with the SRB assay.

We also evaluated migration and invasion of T47D and MDA-MB-231 cells in different concentrations of DHW-208. DHW-208 treatment significantly decreased the migration and invasion in a concentration-dependent manner of T47D and MDA-MB-231 cells compared with the control (Fig. 2), suggesting that DHW-208 was effective in curtailing the migration and invasion of breast cancer cells. Taken together, these results indicate that DHW-208 could inhibit the growth, migration, and invasion of human breast cancer cells.

### DHW-208 induces apoptotic cell death in breast cancer cells

**Fig. 3:**
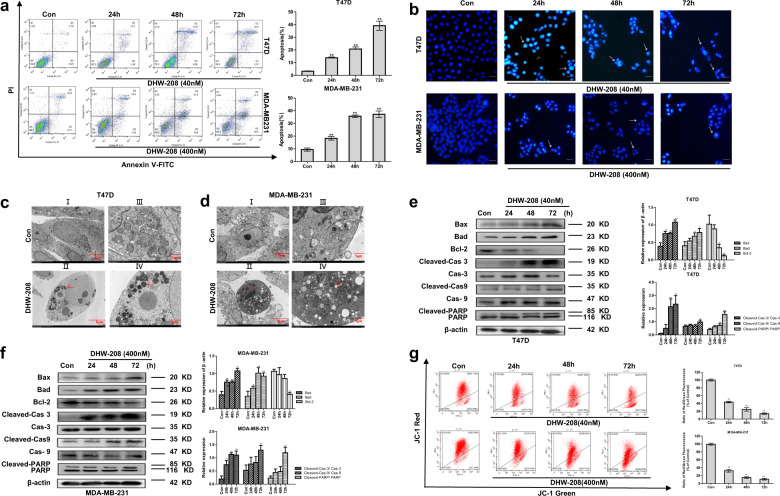
DHW-208 treatment causes cell apoptosis in breast cancer cells. **a** Annexin V-FITC/PI double-staining of cells treated with DHW-208 (40 and 400 nM for T47D and MDA-MB-231 cells, respectively) for the indicated times. The Annexin V-FITC/PI double-staining was quantified and plotted on the right. **b** Changes in various human breast cancer cells treated with DHW-208 (40 and 400 nM for T47D and MDA-MB-231 cells, respectively) visualized by Hoechst 33342 staining (×200 magnification, scale bar = 100 μm). Arrows, apoptotic cells. **c**, **d** Morphologic changes in cells treated with DHW-208 (40 and 400 nM for T47D and MDA-MB-231 cells, respectively) were observed after 48 h by transmission electron microscopy (I, II ×2500 magnification, scale bar = μm [left side], III, IV ×8000 magnification, scale bar = 1 μm [right side]). Red arrow, typical apoptotic micronuclei. **e**, **f** Changes in apoptosis-related proteins in cells treated with DHW-208 (40 and 400 nM for T47D and MDA-MB-231 cells, respectively) for the indicated times by western blot. Bar graphs of the quantitative results were shown right. **g** MMP was detected by JC-1 staining after treatment with DHW-208 (40 and 400 nM for T47D and MDA-MB-231 cells, respectively) for the indicated times. The JC-1 staining was quantified and plotted on the right. Each value is the mean (±SD) from triplicate samples. *p < 0.05, **p < 0.01 vs. control.

Apoptosis is an important way for anticancer drugs to eliminate cancer cells. To evaluate whether DHW-208 is able to induce apoptotic cell death, cells were treated with DHW-208, and Annexin V-positive cells were detected by FACS analysis. As shown in Fig. 3a, DHW-208 induced cell apoptosis in a time-dependent manner in both T47D (from 3.2% to 36.44% at 40 nM DHW-208) and MDA-MB-231 (from 5.89% to 57.09% at 400 nM DHW-208) cells. In addition, Hoechst 33342 staining confirmed the apoptosis by the appearance of condensed chromatin and fragmented nuclei in the cytoplasm (Fig. 3b). Cell ultrastructure <0.2 μm can be observed by transmission electron microscopy, which is considered the gold standard to determine apoptosis. Figure 3c and d reveal that the cells exhibited typical apoptotic features including chromatin condensation and margination at the nuclear periphery after DHW-208 treatment. In Fig. 3c, panels I and III show T47D cells of the control group with clear cell spacing and intercellular connections, but not tight connections. Panels II and IV show T47D cells treated with DHW-208, revealing increased heterochromatin in the nucleus and chromatin condensed into apoptotic bodies. Similar findings were observed in MDA-MB-231 cells (Fig. 3d).

To further clarify the molecular mechanisms of this process, the expression of apoptosis-related proteins in the lysates of DHW-208-treated T47D and MDA-MB-231 cells was examined by western blot analysis. As shown in Fig. 3e and f, the anti-apoptotic protein Bcl-2 was downregulated by DHW-208 in a time-dependent manner. Meanwhile, DHW-208 upregulated the expression of Bax and Bad. The levels of cleaved caspase-3, cleaved caspase-9, and cleaved poly (ADP-ribose) polymerase (PARP) in human breast cancer cells exhibited similar trends. And DHW-208 could reduce the mitochondrial membrane potential in a time-dependent manner (Fig. 3g). These observations indicate that DHW-208 can trigger the activation of the caspase-dependent apoptotic cascade and induce apoptosis of human breast cancer cells through the mitochondrial pathway.

### DHW-208 induces cell cycle arrest in breast cancer cells

**Fig. 4:**
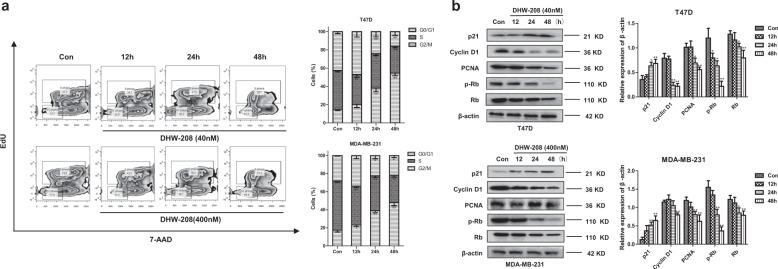
DHW-208 alters cell cycle distribution in breast cancer cells. **a** FACS analysis of the cell cycle distribution after treatment with DHW-208 (40 and 400 nM for T47D and MDA-MB-231 cells, respectively) for 0–48 h. The original histogram plots are shown on the left, and the cell quantification in specific cell cycle phases is shown on the right. **b** Western blot for the levels of cell cycle-related proteins in DHW-208-treated T47D and MDA-MB-231 cells for 0–48 h. Bar graphs of the quantitative results of western blot were shown on the right. Each value is the mean (±SD) from triplicate samples. *p < 0.05, **p < 0.01 vs. control.

To evaluate the mechanism of DHW-208-mediated cell growth inhibition, cell cycle distribution was assessed by FACS analysis. DHW-208 arrested both T47D and MDA-MB-231 cells in G0/G1 phase in a time-dependent manner within 48 h (Fig. 4a). Moreover, the influence of DHW-208 on cell cycle arrest were further verified by Western blot. Consequently, our results supported that DHW-208 played an important role in G0/G1 cell cycle arrest by upregulating expression of p21 as well as downregulating levels of Cyclin D1, PCNA, Rb, and p-Rb proteins in both T47D and MDA-MB-231 cells (Fig. 4b). These results suggest that the anti-proliferation activity of DHW-208 in breast cancer cells is associated with G0/G1 cell cycle arrest.

### DHW-208 inhibits the PI3K/AKT/mTOR-signaling pathway in breast cancer cells

**Fig. 5:**
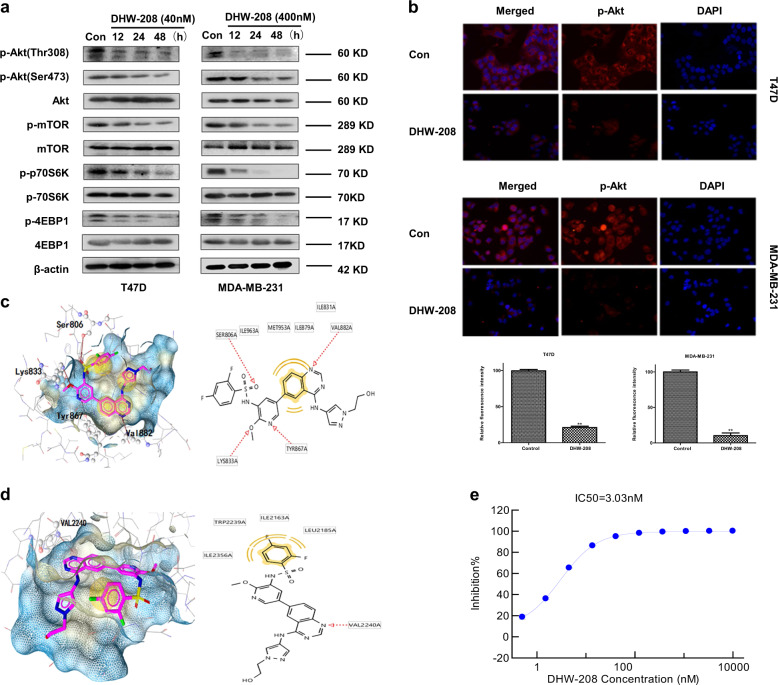
Effect of DHW-208 on the PI3K/AKT/mTOR pathway. **a** DHW-208 suppressed the PI3K/AKT/mTOR-signaling pathway in T47D and MDA-MB-231 cells. Cells were treated with or without DHW208 (40 and 400 nM for T47D and MDA-MB-231 cells, respectively) for the indicated times. PI3K signaling-related protein levels were measured by western blot. **b** Immunofluorescence analysis was performed to determine the effects of DHW-208 on the level of p-AKT (Ser473) in T47D and MDA-MB-231 cells. The cells were treated with DHW-208 (40 and 400 nM for T47D and MDA-MB-231 cells, respectively) for 12 h, and then p-AKT expression was observed under a fluorescence microscope (×400 magnification). Quantification of the fold changes in the levels of p-AKT were shown down. **c**, **d** Three-dimensional (left) and two-dimensional (right) images of molecular docking model for DHW-208 in the PI3K (PDB code: 3l08) **c** and mTOR (PDB code: 4JT6) **d** binding pocket. Interactions are indicated with the following color codes: hydrogen bond acceptor (red arrow), hydrophobic interaction (yellow sphere). **e** The inhibitory curve of DHW-208 on mTOR kinase activity. Each value is the mean (±SD) from triplicate samples. **p < 0.01 vs. control.

To further explore the anticancer mechanism of DHW-208, we evaluated the effects of DHW-208 on the PI3K/AKT/mTOR pathway in breast cancer cells using western blot analysis. MDA-MB-231 and T47D cells were treated with DHW-208 for various times, and the phosphorylation levels of PI3K/AKT/mTOR signaling intermediates including p-AKT, p-mTOR, p-p70S6K, and p-4EBP1 were effectively suppressed (Fig. 5a). Immunofluorescence analysis also confirmed the results. The phosphorylation levels of AKT in breast cancer cells were significantly inhibited by DHW-208 in both T47D and MDA-MB-231 cells (Fig. 5b).

In order to understand the PI3K and mTOR protein-binding modes of DHW-208, we performed molecular docking analysis on the active pocket of the PI3K (PDB code: 3l08) and mTOR (PDB code: 4JT6) crystal structure (Fig. 5c, d). We noted that DHW-208 forms multiple hydrogen bonds with Ser806, Lys833, Tyr867, and Val882. Remarkably, a stable hydrogen bond was established between the sulfonamide oxygen and the hydroxyl group of Ser806 and a stable hydrogen bond was formed between the nitrogen atom on the pyrimidine ring of DHW-208 and the amino group in Val882. DHW-208 could be further stabilized in the PI3K-binding site by hydrophobic interactions of its nonpolar groups with the side chains of Ile963, Met953, Ile879, and Ile831 (Fig. 5c). Thus DHW-208 had substantially strong affinity for the active site of PI3K. The predicted binding mode of DHW-208 on mTOR was shown in Fig. 5d. DHW-208 could bind to mTOR complex and the protein–ligand complex was stabilized by H-bonding and hydrophobic interactions. In our previous study, we found that DHW-208 showed significant kinase inhibitory activity against four main subunits of PI3K^31^. Presently, DHW-208 also showed strong inhibitory activity against mTOR kinase. We examined the in vitro kinase activity of mTOR in the presence of DHW-208, and the results showed that DHW-208 could inhibit the activity of mTOR kinase with the IC_50_ value of 3 nM (Fig. 5e). These results indicated that DHW-208 could be a potential compound targeting both PI3K and mTOR. The above results confirmed that DHW-208 was a dual PI3K and mTOR inhibitor suppressing the PI3K/AKT/mTOR-signaling pathway in breast cancer cells.

### DHW-208 inhibits breast cancer cell growth in vivo

**Fig. 6:**
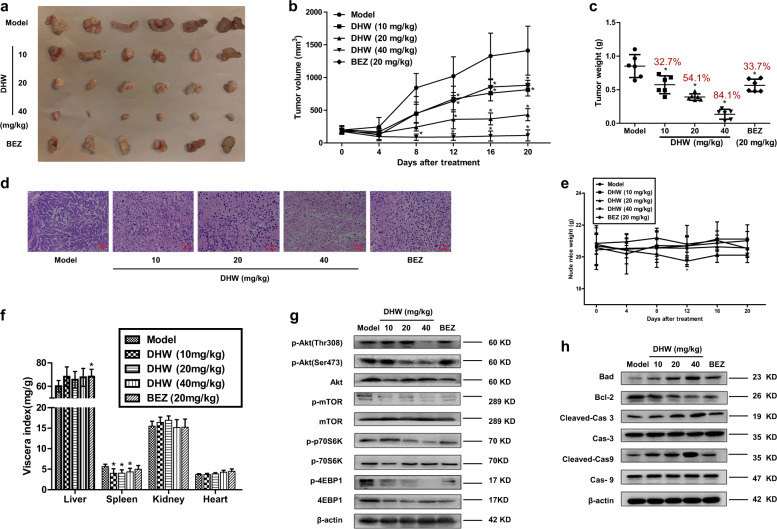
DHW-208 induces a potent antitumor effect in T47D nude mouse xenograft model. **a** Images of resected human breast tumor samples in this study. Control (qd×20, Po, DMSO:PEG300:saline = 1:4:5); DHW-208 (qd×20, Po, 10, 20, and 40 mg/kg, respectively); BEZ235 (qd×20, Po, 20 mg/kg). **b** Body weights of T47D nude mice were measured at the end of the indicated treatment. **c** Average tumor volumes were measured every 4 days. **d** Average tumor weight at the end of the indicated treatment. **e** Representative photomicrographs of H&E staining in T47D xenograft tumors (×400 magnification, scale bar = 100 μm). **f** Viscera index of the main organs isolated from the treated and control mice to evaluate the toxicity of DHW-208. **g**, **h** Effect of DHW-208 on PI3K/AKT/mTOR-signaling proteins **g** and apoptosis-related proteins **h** in tumor tissues was analyzed by western blot (T47D xenograft). Data are shown as mean ± SD (*n* = 6). *p < 0.05 vs. control (Model).

To validate the in vitro results, the antitumor activity of DHW-208 in vivo was further determined in a nude mouse xenograft model. DHW-208 (10, 20, or 40 mg/kg) or BEZ235 (20 mg/kg) was orally administered daily for 20 days. BEZ235 is an imidazoquinoline derivative inhibiting both PI3K and mTOR kinases with good anti-tumor activity orally^32^. Remarkably, tumor growth in the DHW-208-treated mice (10, 20, and 40 mg/kg) was attenuated compared with that in the vehicle-treated mice (Fig. 6a, b). In the T47D cell-implanted xenograft model, the percent reduction in tumor weight relative to the control group was 32.7%, 54.1%, and 84.1% with 10, 20, and 40 mg/kg of DHW-208, respectively, and 33.7% with 20 mg/kg of BEZ235 (Fig. 6c). H&E staining also verified the inhibitory effect of DHW-208 on breast tumor cells (Fig. 6d). No overt toxicity or body weight change was observed with DHW-208 at 10, 20, or 40 mg/kg (Fig. 6e). No obvious differences were observed in the viscera indexes of the heart, liver, and kidneys of the DHW-208-treated group compared to that of control group. However, the viscera index of the spleen was decreased (Fig. 6f).

In addition, the protein levels of p-AKT, p-mTOR, p-70S60K, and p-4EBP1 were all downregulated by DHW-208, which suggests that the PI3K/AKT/mTOR-signaling pathway was inhibited by DHW-208 in the in vivo model (Fig. 6g). The expression levels of proteins related to apoptotic cell death, including Bcl-2, Bax, cleaved caspase-3, and cleaved caspase-9 in the tumor tissues was also consistent with the results in vitro (Fig. 6h). All of these results suggest that DHW-208 exhibits potential anti-breast cancer effects by inhibiting PI3K/AKT/mTOR pathway with low toxicity in vivo.

## Discussion

Breast cancer is one of the most common malignant tumors in women worldwide. To date, there has been no effective treatment strategy for advanced breast cancer with metastasis^3,13^. Thus, the development of new and effective drugs to treat breast cancer is urgently needed. The PI3K/AKT/mTOR-signaling pathway has been well documented as playing a major role in carcinogenesis in breast cancer cells^14,33,34^. In this study, we investigated the mechanism of a dual PI3K and mTOR inhibitor, DHW-208 on the growth of breast cancer cells both in vitro and in vivo. Our study suggests that DHW-208 is able to reduce the growth, migration, and invasion of human breast cancer cells with low toxicity to normal human breast cells.

The PI3K/AKT/mTOR-signaling pathway is an important way in the regulation of cell apoptosis and cell cycle. Our study indicates that DHW-208 could induce cell apoptosis and G0/G1 cell cycle arrest in breast cancer cells. Bcl-2-related death promoter Bad and Bax are important substrates of AKT^35–38^. The Bcl-2 family of proteins include anti-apoptotic proteins such as Bcl-2 and pro-apoptotic proteins such as Bad and Bax^39,40^. Apoptosis is controlled by the balance between pro-apoptotic and anti-apoptotic proteins^41–43^. Downregulation of Bcl-2 and upregulation of Bax and Bad were observed in lysates of T47D and MDA-MB-231 cells exposed to DHW-208. Increased pro-apoptotic protein levels or decreased anti-apoptotic protein levels could result in reduced or abolished mitochondrial membrane potential, thereby releasing death factors and promoting the activation of caspase-9^44–46^. Activated caspase-9 can activate procaspase-3 downstream, which can further cleave PARP, leading to the apoptotic cascade^13,42,47^. Activated caspase-3 also induces the downstream mitochondrial apoptotic pathway, thereby inhibiting AKT^43,48–51^. Time-dependent cleavage of caspase-3, caspase-9, and PARP was observed in the DHW-208 treatment cells indicating that DHW-208 can reduce activity of the caspase (intrinsic) pathway. The mitochondrial membrane potential was decreased after treatment with DHW-208. These observations indicate that the mitochondrial pathway is the main mechanism underlying the apoptosis of breast cancer cells induced by DHW-208. Our study indicated that DHW-208 arrested the growth of T47D and MDA-MB-231 cells at the G0/G1 phase of the cell cycle. AKT could directly activate mTOR, and activated mTOR could activate p70S6K and 4EBP1 downstream, both of which could accelerate the transcription of mRNA required for cell cycle development^47,52^. Therefore, our results suggest that the anti-proliferation activity of DHW-208 in breast cancer cells is associated with G0/G1 cell cycle arrest, which is achieved by regulating the protein expression levels in PI3K/AKT/mTOR.

It has been shown that activation of the PI3K/Akt/mTOR pathway plays crucial roles in cancer development and progression. Activated PI3K/Akt/mTOR pathway have been documented in breast cancers. DHW-208 remarkably suppressed the activation of AKT, mTOR, p70S6K, and 4EBP1 in breast cancer cell T47D and MDA-MB-231, thus inhibited PI3K/Akt/mTOR pathway. Moreover, molecular docking results showed that DHW-208 had strong affinity for PI3K in its active site and could bind to mTOR complex by H-bonding and hydrophobic interactions. In our previous study, we discovered that DHW-208 showed significant kinase inhibitory activity against four main subunits of PI3K. Presently, DHW-208 also showed strong inhibitory activity against mTOR kinase. These results confirm that DHW-208 is a dual inhibitor of PI3K and mTOR, and suppress the growth of human breast cancer cells by targeting the PI3K/AKT/mTOR pathway.

We found that DHW-208 showed not only tumor selectivity for breast cancer cells in vitro but also relatively weak cytotoxicity and high efficiency in vivo. Compared with PI3K inhibitor BEZ235, DHW-208 exhibited the more potent growth inhibition of tumor growth in xenograft model. In the DHW-208-treated group, the viscera index of the spleen decreased compared with that of the control group. The spleen is the largest peripheral immune organ and plays an important role in resisting the invasion of harmful organisms and changes from the external environment^53,54^. The effect of the drug on the spleen needs to be explored in future experiments. Consistent with the in vitro results, DHW-208 treatment inhibited the expression/phosphorylation of key proteins in the PI3K/AKT/mTOR-signaling pathway compared with those of the control mice and also induced apoptosis in vivo. Therefore, DHW-208 was an effective and hypotoxic compound and inhibited the growth of human breast cancer cells via the PI3K/AKT/mTOR-signaling pathway.

In conclusion, DHW-208 is a dual inhibitor of PI3K and mTOR that can suppress the growth of human breast cancer cells by inhibiting the PI3K/AKT/mTOR-signaling pathway. Our studies indicate that DHW-208 could be an effective anticancer drug for the treatment of breast cancer.
