# Editorial Expression of Concern: A novel 4-aminoquinazoline derivative, DHW-208, suppresses the growth of human breast cancer cells by targeting the PI3K/AKT/mTOR pathway

**DOI:** 10.1038/s41419-025-07539-7

**Published:** 2025-03-27

**Authors:** Shu Wang, Yingshi Zhang, Tianshu Ren, Qiong Wu, Hongyuan Lu, Xiaochun Qin, Yuyan Liu, Huaiwei Ding, Qingchun Zhao

**Affiliations:** 1 Department of Life Science and Biochemistry, Shenyang Pharmaceutical University, 110016 Shenyang, China; 2 Department of Pharmacy, General Hospital of Northern Theater Command, 110840 Shenyang, China; 3 Key Laboratory of Structure-Based Drug Design and Discovery of Ministry of Education, Shenyang Pharmaceutical University, 110016 Shenyang, China

Editorial Expression of Concern to: *Cell Death & Disease* 10.1038/s41419-020-2690-y, published online 30 June 2020

The Editors would like to alert the readers that concerns have been raised regarding the images presented in Fig. 2:

- Three pairs of images in Fig. 2b and c appear to overlap;
- A number of images in Fig. 2b and c appear highly similar to those in other publications, two of which were under consideration within a similar time frame as this article [1, 2].

The authors have provided the original image files, but have been unable to sufficiently address the image similarity with other publications. Readers are therefore advised to interpret these results with caution.

Shu Wang has not explicitly stated whether they agree to this Editorial Expression of Concern. None of the other authors have responded to any correspondence from the publisher about this Editorial Expression of Concern.
